# Vessel Noise Affects Beaked Whale Behavior: Results of a Dedicated Acoustic Response Study

**DOI:** 10.1371/journal.pone.0042535

**Published:** 2012-08-03

**Authors:** Enrico Pirotta, Rachael Milor, Nicola Quick, David Moretti, Nancy Di Marzio, Peter Tyack, Ian Boyd, Gordon Hastie

**Affiliations:** 1 SMRU Ltd, New Technology Center, North Haugh, St Andrews, Fife, United Kingdom; 2 Sea Mammal Research Unit, Scottish Oceans Institute, University of St Andrews, St Andrews, United Kingdom; 3 School of Marine Science and Technology, Newcastle University, Newcastle upon Tyne, United Kingdom; 4 School of Biology, Scottish Oceans Institute, University of St Andrews, St Andrews, Fife, United Kingdom; 5 Naval Undersea Warfare Center Division, Newport, Rhode Island, United States of America; 6 Biology Department, Woods Hole Oceanographic Institution, Woods Hole, Massachusetts, United States of America; Texas A&M University-Corpus Christi, United States of America

## Abstract

Some beaked whale species are susceptible to the detrimental effects of anthropogenic noise. Most studies have concentrated on the effects of military sonar, but other forms of acoustic disturbance (e.g. shipping noise) may disrupt behavior. An experiment involving the exposure of target whale groups to intense vessel-generated noise tested how these exposures influenced the foraging behavior of Blainville’s beaked whales (*Mesoplodon densirostris*) in the Tongue of the Ocean (Bahamas). A military array of bottom-mounted hydrophones was used to measure the response based upon changes in the spatial and temporal pattern of vocalizations. The archived acoustic data were used to compute metrics of the echolocation-based foraging behavior for 16 targeted groups, 10 groups further away on the range, and 26 non-exposed groups. The duration of foraging bouts was not significantly affected by the exposure. Changes in the hydrophone over which the group was most frequently detected occurred as the animals moved around within a foraging bout, and their number was significantly less the closer the whales were to the sound source. Non-exposed groups also had significantly more changes in the primary hydrophone than exposed groups irrespective of distance. Our results suggested that broadband ship noise caused a significant change in beaked whale behavior up to at least 5.2 kilometers away from the vessel. The observed change could potentially correspond to a restriction in the movement of groups, a period of more directional travel, a reduction in the number of individuals clicking within the group, or a response to changes in prey movement.

## Introduction

With increases in propulsion, gross tonnage, and vessel densities [1], [2], shipping traffic is believed to be a major contributor to the continuing rise of noise in the ocean [1], [3], [4]. This has led to concerns about the potential impacts on marine fauna, especially marine mammals (e.g. [3], [5]). Baleen whales, which use low frequency sound, are expected to be most vulnerable to the relatively low frequencies of noise associated with shipping [3], [6]. However, changes in shipping are likely to be associated with increased levels of broadband noise (e.g. due to propeller cavitation resulting from higher vessel speeds; [7]). Energy is thus introduced at higher frequencies, overlapping with toothed whale vocalizations and hearing sensitivity, with potential behavioral or physiological consequences (e.g. [8]–[11]).

Beaked whales are deep-diving odontocetes, which forage regularly at depths of more than 1000 m for periods of more than 1 h [12]. Evidence based upon the occurrence of strandings of beaked whales in association with military mid-frequency sonar suggests that they are particularly susceptible to anthropogenic noise (e.g. [13]–[16]). It is therefore important to consider the effects of common noise sources like shipping [10] on these acoustically sensitive species.

This study aimed to measure behavioral responses of Blainville’s beaked whales (*Mesoplodon densirostris*) to vessel noise. As beaked whales are a visually cryptic species, passive acoustic techniques [17], [18] were used to measure behavior. Blainville’s beaked whales produce short (∼250 µs) echolocation clicks in a narrow beam of about 14° [19] and with a frequency range from 25 to 55 kHz, which are associated with foraging [17], [20], and these can be detected and localized using hydrophone arrays. The study was carried out in the Tongue Of The Ocean (Bahamas), where a bottom mounted hydrophone array allowed the detection and localization of individual beaked whale groups [21]. A series of exposure trials involving targeting whale groups with intense (∼206 dB re 1 µPa at 1 m) vessel-generated noise were carried out to assess whether the propulsion sound from a vessel influenced Blainville’s beaked whale foraging behavior at depth.

## Materials and Methods

### 2.1 Ethics Statement

The research was conducted under permits for marine mammal research issued by the U.S. National Marine Fisheries Service to John Boreman (Permit #1121–1900) and to Peter Tyack (Permit #981–1578), and issued by the Government of the Bahamas to the Bahamas Marine Mammal Research Organisation (Bahamas permit #01/09) and Ian Boyd (Bahamas permit #02/07 and #02/08). This study was carried out in strict accordance with the US Animal Welfare Act following the relevant recommendations of the Guide for the Care and Use of Laboratory Animals of the National Institutes of Health. The study was approved by the Woods Hole Oceanographic Institution and the Bahamas Marine Mammal Organisation Institutional Animal Care and Use Committees and the Animal Welfare and the Ethics Committee of the University of St Andrews.

### 2.2 Background

**Figure 1:**
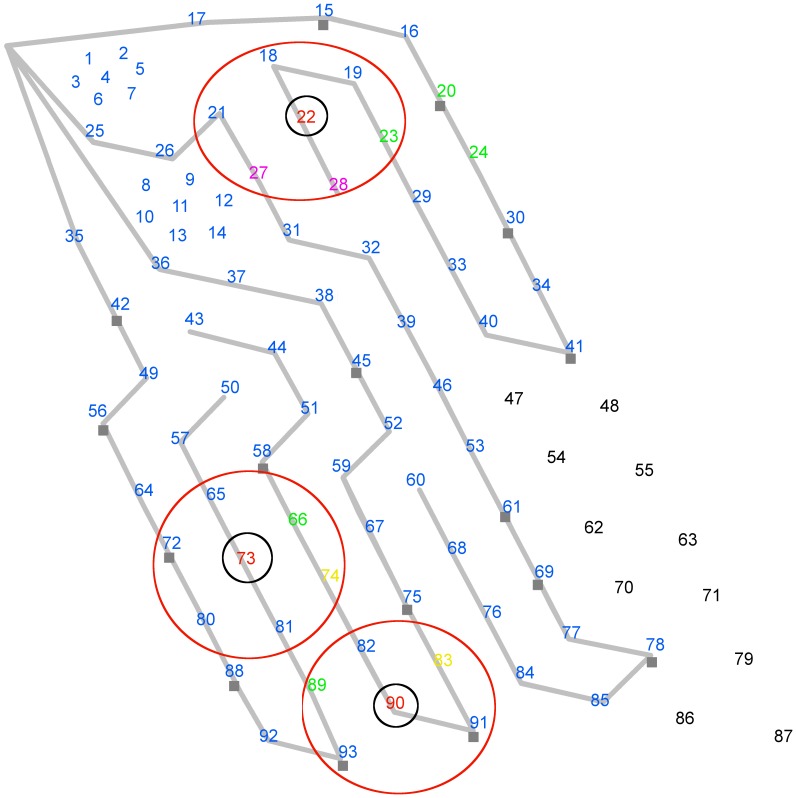
Schematic representation of the hydrophone array (screen-shot from the software MMAMMAL). Circled in black are three primary hydrophones that are recording the presence of three distinct beaked whale groups. Circled in red are the hydrophones within a radius of 3704 m (2 Nm) around each primary hydrophone.

The exposure trials were conducted in September 2007 at the same time as a research project studying the effects of Mid-Frequency Active Sonar on beaked whales [22]. The study took place in the Tongue Of The Ocean, a deep water basin located to the east of Andros Island (Bahamas). In the Tongue Of The Ocean, beaked whale vocalizations were monitored through an array of seabed-mounted hydrophones at the Atlantic Undersea Test and Evaluation Center (AUTEC). These are operated by the U.S. Navy to track the movements of sound-producing vessels in the area [21], [23]. AUTEC includes 82 permanent hydrophones bottom-mounted at depths ≤2000 m over an area of 1124 km^2^. Two original tight clusters (1–7 and 8–14) in the Northwest corner of the range included wideband hydrophones that were approximately 1850 m apart, while more recently installed hydrophones (15–93) were approximately 3700 m apart (Figure 1). Because of its frequency and spacing characteristics, the array could be used to detect the loud (>200 dB re µPa at 1 m; [17]) clicks of Blainville’s beaked whales present both within the area of the hydrophone array and adjacent to the edges of the array [21], [22]. Raw acoustic data were cabled back to shore, where all detections of marine mammal vocalizations were recorded. Beaked whale echolocation clicks were generally detected at distances of up to 6500 m, usually when an animal was pointing within 30 degrees of the hydrophone [19], and several groups could be detected simultaneously on the range.

The hydrophones at AUTEC are arranged in hexagonal arrays surrounding a center hydrophone. A sound source like a foraging beaked whale is detected on multiple hydrophones and can be tracked using a hyperbolic multilateration algorithm [24] that requires four hydrophones to determine the source position in 3 dimensions or three hydrophones if depth is known. Blainville’s beaked whales emit chains of echolocation clicks at a rate of approximately 3 per second [17], but due to their narrow beam pattern [18] detecting clicks on at least three hydrophones is challenging and localization to a precision that is greater than the distance between the hydrophones is generally not possible. Associating clicks is equally difficult as they are highly coherent, making isolation of a single click in a long chain of clicks across multiple hydrophones extremely challenging.

Although we could not track each group’s movement, we were able to estimate the position of the animals by looking at the hydrophone that was consistently detecting their clicks, with an uncertainty about their true location on the array of approximately 3700 m around that hydrophone. On average, animals dive in groups of 2–3 and produce multiple echolocation clicks when foraging begins, usually at depths >200 m. It was assumed that at least some of the clicks from the groups were detected with certainty when these were foraging within the field of sensors [25].

### 2.3 Data Collection

**Figure 2:**
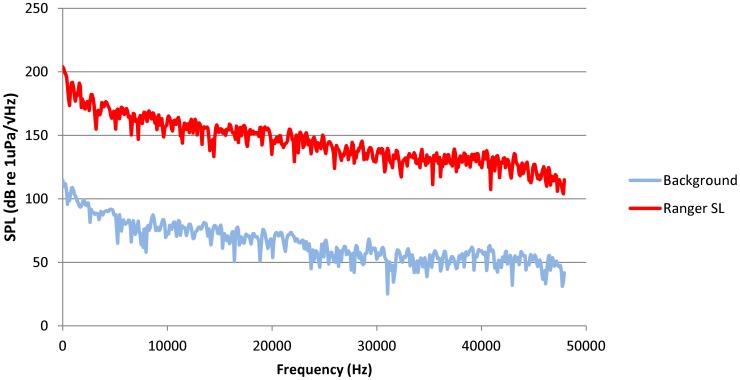
Predicted spectrum of source vessel noise expressed in units of power spectral density. The noise signature is dominated by low frequency noise (<10 kHz) but has high levels across the frequency range of the recordings.

Real time monitoring of the range hydrophone array was used to guide the source vessel (MV Ranger) used in the experiments to vocalizing beaked whales. The vessel maneuvered as close as possible to the estimated location of the whales and waited dead in the water (DIW), until the completion of a surface-dive cycle. Approximately 3–5 min after the re-start of vocalizations (and when the shore based monitoring reported reliable click detection), the source vessel switched to full power for 1–2 minutes. After this, it returned to being DIW for 2 minutes. This procedure was repeated up to six consecutive times during a single foraging dive of the focal group, and it was designed to expose the whale group to noise whilst being able to verify when the whales ceased vocalizing. Adjustments to vessel heading were made to keep the vessel stationed close to the hydrophone with the strongest signals from the whales. Once confirmed that the whales had ceased vocalizing and completed a dive cycle, the source vessel ceased the trial. The noise produced by the source vessel during one of the trials was estimated from sound data recorded by the closest hydrophone using a 20 log(R) estimation (where R is distance in meters); this showed that the vessel was relatively loud at full power (Broadband RMS Source level (0.1–48 kHz) = 208.66 dB re 1 µPa at 1 m; Figure 2).

### 2.4 Data Processing and Statistical Analysis

Two behavioral metrics were recorded for each whale group: the foraging duration (defined as the length of time in minutes over which sequences of clicks corresponding to a synchronized foraging dive from a group were detected), and the number of times the primary hydrophone of the vocalizing group changed while the whales were foraging. The program MMAMMAL (U.S. Navy; [26]) was used to identify the hydrophones on which Blainville’s beaked whale clicks were present. A binary spectrogram display for each hydrophone was used to mark the beginning and end of each bout of vocalizations, and to estimate the foraging duration. In the binary spectrogram, FFT bins were set to either ‘0’ or to ‘1’ if they passed a threshold level based on the background noise. The default color for all detections on the MMAMMAL display is black. If a click is detected, i.e. at least 10 bins of the FFT are above threshold (or set to ‘1’), the click is colored red. In general, more than one hydrophone detected the clicks from the group (i.e. were ‘active’). Within a group of active hydrophones a primary hydrophone, defined as the hydrophone most consistently detecting clicks (i.e. continuously displaying red clicks), could be identified (Figure 1). Visual assessment of the binary spectrograms of active hydrophones was used to confirm the primary hydrophone. This procedure could not be used to calculate the exact movement path of the groups, but it was sufficiently accurate to investigate their general orientation. The primary hydrophone within a group could shift during the course of the foraging bout depending on the orientation of the whales to the hydrophones. These changes were thus used to define the second measurement: the number of times the primary hydrophone of the vocalizing group changed across the foraging duration. For each group, the overall number of hydrophones available in the area was also computed as the sum of the hydrophones within a radius of 3704 m (2 Nm) around each primary hydrophone during the clicking bout (Figure 1). This number was used in the analysis to account for different densities of hydrophones in different portions of the array. It was not possible to determine click rates or click frequency before, during and after each vessel sound exposure, because clicks were not discernible from background noise when the vessel was at full power. A total of 16 trials were completed on 16 whale groups (hereafter ‘treatments’). In addition, 13 other whale groups that were present elsewhere on the range during the treatments were included in the analysis (hereafter ‘non-focal groups’), because they were exposed to noise, at relatively large distances from the source ship. Finally, 29 groups were selected from time periods when no treatment or other boat activity was present on the range (hereafter ‘non-concurrent controls’). In order to avoid any confounding effect of diurnal patterns in foraging durations and whale behavior, one non-concurrent control at the same time of the day was selected for each whale group present on the range during the treatments. In some instances, more than one beaked whale group was clicking at the same time in the same region of the hydrophone array. Because the foraging duration was visually evaluated from the MMAMMAL display, the convergence of more groups on the same primary hydrophones made it impossible to identify with precision the beginning and end of each clicking bout. Therefore, clicking bouts longer than 40 minutes were excluded, as it was assumed that a longer duration corresponded to vocalizations from more than one group overlapping on the same primary hydrophone. The threshold of 40 minutes was chosen on the basis of the maximum vocal phase duration reported for Blainville’s beaked whales (43.13 min, [27]; 33.1 min, [12]). Three non-focal groups showing a clicking bout that exceeded the 40-minute limit were excluded and, to avoid unbalanced data, the three corresponding non-concurrent controls were also removed. The final data set included 16 treatments and 10 non-focal groups (Table 1), as well as 26 non-concurrent controls.

**Table 1:** Summary of the exposed whale groups used in the analysis.

Date	Time	Exposure
13/09/2007	12∶58	targeted
13/09/2007	15∶15	targeted
13/09/2007	17∶09	targeted
13/09/2007	13∶11	on the range
13/09/2007	17∶01	on the range
14/09/2007	08∶40	targeted
14/09/2007	10∶41	targeted
14/09/2007	11∶22	targeted
14/09/2007	11∶55	targeted
14/09/2007	08∶53	on the range
14/09/2007	10∶45	on the range
15/09/2007	11∶34	targeted
15/09/2007	13∶25	targeted
15/09/2007	13∶39	targeted
15/09/2007	14∶21	targeted
15/09/2007	15∶48	targeted
15/09/2007	13∶18	on the range
15/09/2007	14∶01	on the range
20/09/2007	11∶31	targeted
20/09/2007	11∶41	on the range
26/09/2007	08∶39	targeted
26/09/2007	11∶29	targeted
26/09/2007	14∶01	targeted
26/09/2007	08∶35	on the range
26/09/2007	11∶51	on the range
26/09/2007	14∶16	on the range

These include ‘targeted’, i.e. directly approached by the Ranger, and ‘on the range’ groups, i.e. the others that were present elsewhere on the range during the exposures.

To assess whether vessel noise influenced the behavior of these beaked whale groups, the two behavioral metrics described above (foraging duration and number of primary hydrophone changes) were used as response variables within a Generalized Linear Modeling (GLM) framework [28].

To evaluate the effect of the distance between the source vessel and the whale group on the foraging duration of the groups exposed to the trials, a Gaussian GLM was fitted in R version 2.9.2 [29]. Distances in kilometers were estimated using the ‘map’ function in MMAMMAL and were taken as the straight line distance between the hydrophone over which the source vessel was located, and the average position of each whale group, calculated using the different primary hydrophones identified in each clicking bout. A stepwise procedure based on the Akaike Information Criterion (AIC; [30]) was then used for model selection and the significance of the predictor was finally assessed using the *p*-value resulting from a chi-square approximation (function Anova in the car library in R; [31]).

A Poisson-based GLM was then used to model the effect of the distance from the source vessel on the number of hydrophone changes (i.e. the second metric computed for each group). The foraging duration was also included as a covariate in the models because a longer clicking bout gave the whales more chance to move between primary hydrophones. The overall number of hydrophones within a radius of 3704 m (2 Nm) around each primary hydrophone that was active during the foraging duration was used as an offset in all the models. Models were selected by a stepwise process based on the AIC, followed by a chi-square test to assess the significance of the retained predictors.

Because the exposures could have affected the whales irrespective of their distance from the sound source, the 26 groups recorded at times when there were no exposures or when there was no other boat activity in the area (non-concurrent controls) were then included in the analysis. We defined a categorical variable for exposure: the 26 groups present on the array during the trials (treatments and non-focal groups) were classified altogether as ‘exposed’, while the 26 non-concurrent control groups were classified as ‘non-exposed’. A Gaussian GLM was used to test the difference in foraging duration between exposed and non-exposed groups, while a Poisson-based GLM was used for the difference in the number of primary hydrophone changes.

## Results

**Figure 3:**
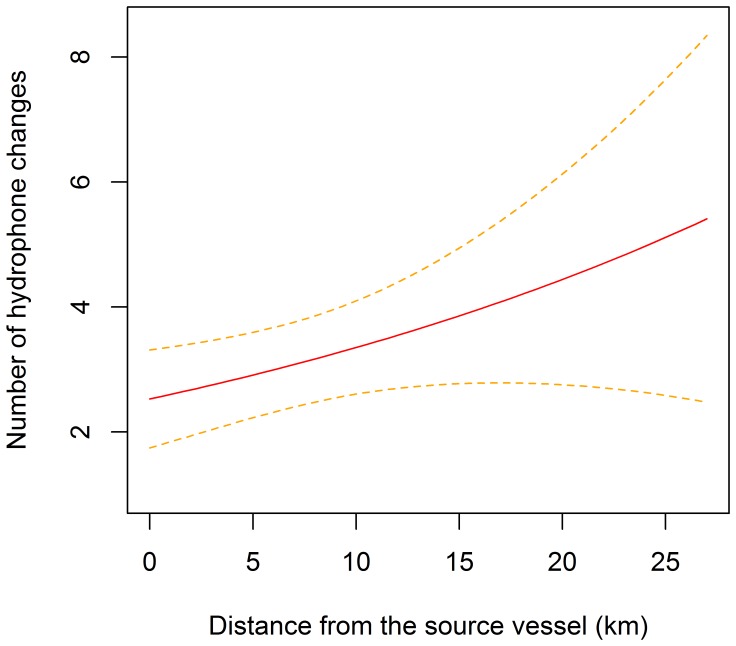
Distance from the source vessel and number of hydrophone changes performed during a foraging bout. This is the relationship predicted by the Generalized Linear Model. Distances are expressed in kilometers.

The foraging duration of beaked whale groups present on the range during the exposures varied between 21.2 and 38.0 min with a mean of 29.4 min (standard deviation, SD: 4.5 min). The distance from the source vessel had no significant effect on the foraging duration (*p* = 0.27). The number of changes of primary hydrophone across the duration of each clicking bout varied between 0 and 6, with a mean of 3 (SD: 2), for the groups that were recorded on the range during the exposures. The distance from the source vessel was a significant predictor of the number of hydrophone changes observed (*p*<0.05). Specifically, the number of hydrophone changes per group decreased by a factor of 0.97 for each kilometer decrease in distance from the sound source. A group at the maximum distance (27 km) from the source vessel made 5.4±2.9 changes within a foraging bout, while a group at zero distance made 2.5±0.8 changes (the predicted relationship is represented in Figure 3). Foraging duration did not contribute significantly to the model, suggesting that the number of hydrophone changes shown by each group did not depend on the duration of the clicking bout.

**Figure 4:**
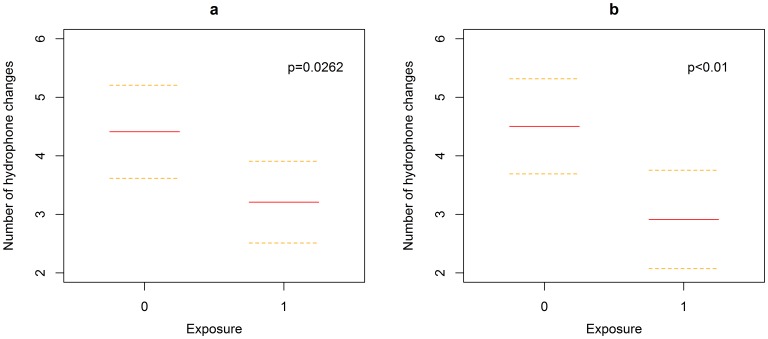
Number of hydrophone changes modeled as a function of the exposure. In a) 0 corresponds to the non-concurrent controls (i.e. not exposed to the sound source), and 1 to the treatments and the non-focal groups (i.e. all the groups present on the range during the trials). In b) 0 refers to the non-concurrent controls, and 1 to the treatments (i.e. the groups targeted by the trials). Dashed lines represent the 95% confidence intervals.

When the data set were extended to include the 26 groups recorded in periods during which no exposures or other boat activity was present on the range (non-concurrent controls), the foraging duration ranged between 19.2 and 40.0 min with a mean of 28.6 min (SD: 5.1), and the number of hydrophone changes varied between 0 and 9, with a mean of 4 (SD: 2). The inclusion of these non-concurrent controls showed that the number of hydrophone changes was significantly reduced (*p = *0.0262) in the groups exposed to the vessel noise at any distance from the source vessel (i.e. the treatments and the other groups present during the exposures) (Figure 4a). The exposed groups made 3.2±0.7 hydrophone changes, compared with the 4.4±0.8 changes by the non-concurrent groups (i.e. 27% decrease on average). Again, the foraging duration did not influence the number of hydrophone changes. We found no difference in the foraging duration between non-concurrent controls and groups present on the range during the exposures.

Comparing the non-concurrent controls with the treatments, while excluding the concurrent non-focal groups from the analysis, gave a highly significant effect of ship noise on the number of hydrophone changes within a clicking bout (*p*<0.01). The magnitude of the effect was also larger in that the groups targeted by the exposures had 2.8±0.8 hydrophone changes, i.e. 35% fewer changes when compared with the non-concurrent controls (4.4±0.8 changes; Figure 4b). Foraging duration had no significant effect upon the number of hydrophone changes.

Finally, we calculated the maximum distance from the source vessel at which we were able to detect a significant change in the whales’ behavior. This was defined as the value of distance after which the upper confidence limit of the estimated relationship between the distance and the number of hydrophone changes overlapped with the lower confidence limit of the number of hydrophone changes made by the non-concurrent control groups, and corresponded to 5.2 km.

All the models were assessed to verify that the underlying assumptions were not violated. The results of this assessment are summarized in Table 2. It is possible that some individual whales were repeatedly exposed to the trials; nevertheless, the analysis should not be affected, as model residuals were in all cases found to be independent through the Durbin-Watson test.

**Table 2:** Results of the assessment of the models used in the analysis.

Gaussian Models			
	Shapiro-Wilk test (Normality)	Breusch-Pagan test (Heteroscedasticity)	Durbin-Watson test (Independence)
*Foraging duration ∼ Distance*	*p* = 0.86	*p* = 1.00	*p* = 0.33
*Foraging duration ∼ Exposure*	*p* = 0.45	*p* = 0.23	*p* = 0.69
**Poisson Models**			
	**Deviance chi-square distributed**	**ACF plot (Independence)**	**Scale parameter (Overdispersion)**
*# hydrophone changes ∼ Distance*	*p* = 0.67	✓	0.6
*# hydrophone changes ∼ Exposure*	*p* = 0.56	✓	0.8
*# hydrophone changes ∼ Exposure (treatments vs. non-concurrent controls)*	*p* = 0.60	✓	0.8

No evidence was found against modeling assumptions.

## Discussion

The results of this study suggest that vessel noise has a significant effect on the movement behavior of Blainville’s beaked whales while they are foraging. Although we did not measure any significant change in the duration of foraging periods, we found a significant effect on the number of changes in the primary hydrophone on which the whales were located within the foraging periods. Furthermore, for those groups that were exposed, there was a positive relationship between the number of hydrophone changes with distance from the vocalizing group to the source vessel. The exposed groups had an average distance from the noise source of 7 km and a maximum distance of 27 km, suggesting that particularly loud vessel noise could have an effect on beaked whale behavior even at relatively large distances. We used a conservative approach and calculated the maximum distance at which we were able to detect a significant behavioral response to be 5.2 km.

Using relatively rudimentary sound propagation models (e.g. 20 log(Range)), we estimate that broadband received levels at whale groups, at the distances measured in this current study (0–27 km), would be approximately 209–120 dB re 1 µPa. Therefore, the estimated received level at 5.2 km is 135 dB re 1 µPa. Ambient noise levels in this region have been measured at between approximately 10 and 70dB re 1 µPa/√Hz (up to 45 kHz) [32]; it should be noted that background noise levels reported in this current study were made when the playback vessel was present (but was stationary) and are therefore not directly comparable to ambient noise levels in Ward et al.’s [32] study. The source level of the Ranger was relatively high, with a spectrum that was comparable to the few published records for cargo ships [3], but with elevated components at high frequencies [7], [10]. The pattern of exposure was also unusual, since a normal ship is not expected to alternate periods at full power to pauses DIW. This would make the exposure settings comparable with the short passage of a large ship moving at high speeds [7], [10]. Despite the relatively unique features of the source vessel noise, it is possible to make some broad generalizations about the observed changes in behavior and the results provide important insights into how the species responds to intense vessel noise.

The response we have described differs from that to the loud (210 dB), short (1 sec), and band-limited (∼3.25–3.75 kHz) sonar, pseudo-random noise and killer whale signals that was found by Tyack et al. [22] in that the exposed whales did not prematurely cease foraging. This difference might result from the different sound characteristics to which the animals were exposed. For example, the tonal nature of a sonar or killer whale sound may cause a more dramatic response than broadband noise [10]; this has been observed previously in right whales [33]. In addition, the source vessel used for the exposures was often present on the range when intense sonar sounds were absent. The vessel noise by itself may thus evoke a cautious response, but less cautious than sounds of sonar or a simulated predator.

Our results differ from the limited previous evidence of beaked whale responses to vessel noise in which a tagged Cuvier’s beaked whale exhibited a shorter dive (15 min shorter than the mean. of the unexposed dives) and a shorter vocal period during a dive with elevated noise from a passing ship [10]. Direct comparisons of responses between different species are difficult, and factors such as habituation to the noise of the experimental vessel, the level of exposure, or the location [34] could all contribute to this difference. However, given that there had only be a single observation of a response before the present study, further detailed analysis of the differences are not likely to be informative.

The pattern of change of primary hydrophones in relation to exposure could be interpreted as a restriction in the movement of the groups caused by exposure to ship noise. This type of response has been shown previously in Finley et al. [35], who found narwhals reduced their movements in response to the noise of ice-breaking ships. Alternatively, given the narrow beam pattern of beaked whale clicks [19], a reduction in the number of primary hydrophones could indicate a period of more directional travel by the whales; a feature that was exhibited by beaked whales in response to simulated sonar and killer whale playbacks [22]. The response could also indicate a reduction in the number of individual whales clicking within the group. In addition, the whales could have been responding to changes in the movement of prey caused by the ship noise. Nevertheless, it is likely that this result is indicative of vessel noise interfering with the foraging efficiency of the exposed individuals. Prolonged dives at depth are energetically expensive [12], and an alteration of the animals’ behavior in the foraging patch could reduce the food intake and subsequent energy gain associated with their foraging bouts during exposure [36], [37]. The concern about such behavioral changes is thus likely to be chronic rather than acute, with a progressive reduction of condition associated with the cumulative behavioral disruption. Such energetic deficiencies have the potential to lead to impacts on individual survival and reproductive capability and, ultimately, could lead to population decline [3], [34].

As an aside, our study also has interesting implications on the experimental design of controlled exposure experiments [38]: the use of a noisy vessel to approach the animals during tagging or monitoring operations might significantly alter the natural behavioral pattern of the whales and thus confound the results of a playback experiment. The number of boats present in the area and the amount of noise that these introduce in the environment should therefore be used as a covariate in experimental studies in order to be able to discern between the effects of the playbacks and the confounding effects of boat noise.

In conclusion, despite the differences in the nature of the response found between this and previous studies, our work confirms the particular sensitivity of beaked whale behavior to acoustic exposure [22], [39]. We have shown that broadband vessel noise causes significant changes in the natural foraging behavior of Blainville’s beaked whales, with evidence that, for the given scenarios, it significantly affected individuals up to at least 5.2 kilometers away from the sound source. Our results thus strengthen the conclusion by Aguilar de Soto et al. [10] that this source of noise pollution might also have significant effects on odontocete species.
